# Fatty-Acid-Binding Proteins: From Lipid Transporters to Disease Biomarkers

**DOI:** 10.3390/biom13121753

**Published:** 2023-12-06

**Authors:** Shabarni Gaffar, A Sayyidatina Aathirah

**Affiliations:** 1 Graduate School, Padjadjaran University, Bandung 40132, Indonesia; asayyidatinaa@gmail.com; 2 Department of Chemistry, Faculty of Mathematics and Natural Sciences, Padjadjaran University, Sumedang 45363, Indonesia

**Keywords:** FABPs, biomarkers, overexpression, diagnosis

## Abstract

Fatty-acid-binding proteins (FABPs) serve a crucial role in the metabolism and transport of fatty acids and other hydrophobic ligands as an intracellular protein family. They are also recognized as a critical mediator in the inflammatory and ischemic pathways. FABPs are found in a wide range of tissues and organs, allowing them to contribute to various disease/injury developments that have not been widely discussed. We have collected and analyzed research journals that have investigated the role of FABPs in various diseases. Through this review, we discuss the findings on the potential of FABPs as biomarkers for various diseases in different tissues and organs, looking at their expression levels and their roles in related diseases according to available literature data. FABPs have been reported to show significantly increased expression levels in various tissues and organs associated with metabolic and inflammatory diseases. Therefore, FABPs are a promising novel biomarker that needs further development to optimize disease diagnosis and prognosis methods along with previously discovered markers.

## 1. Introduction

Fatty-acid-binding proteins (FABPs) are a class of low-molecular-weight intracellular proteins that play a role as a transporter by binding to hydrophobic ligands, typically fatty acids, with different affinities, and are involved in the metabolism of these fatty acids (FAs). These hydrophobic ligands include, but are not limited to, saturated fatty acids, monounsaturated fatty acids (MUFAs), polyunsaturated fatty acids (PUFAs), eicosanoids, and other lipids. FABPs have been shown to be important in modulating lipid metabolism, gene regulation, and signal transmission [1,2]. They have been thought of as the key mediators in the metabolic and inflammatory processes [1,3,4].

**Figure 1:**
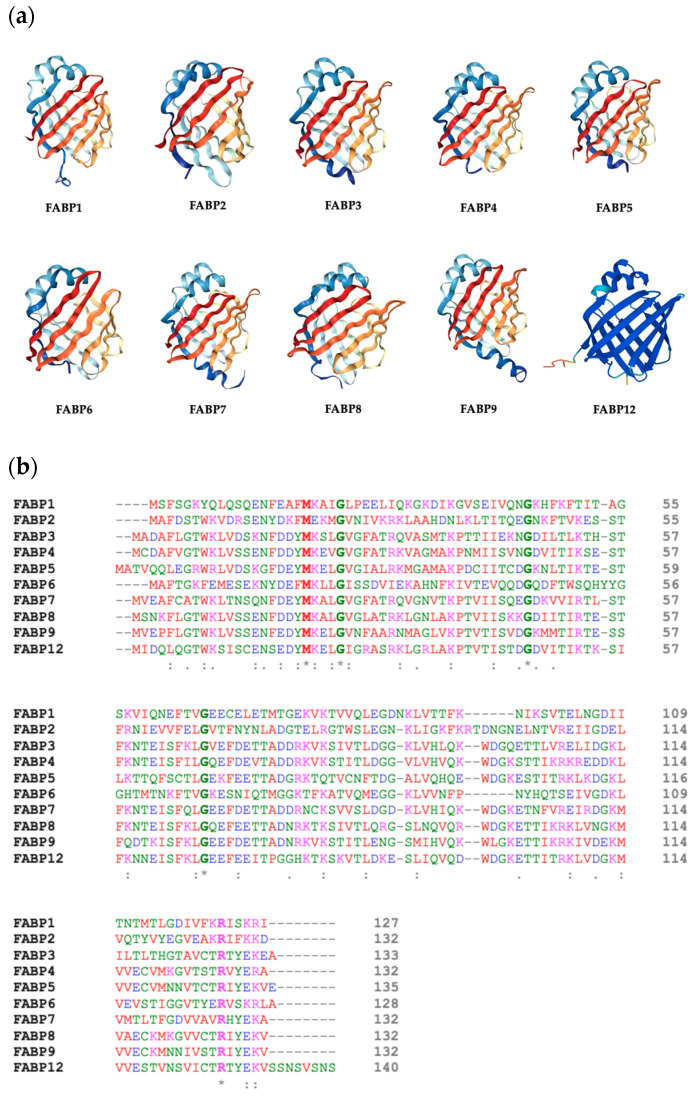
(**a**) The 3D structure of human FABPs. The 3D structures of FABP1–FABP9 were obtained from Protein Data Bank (PDB, https://www.rcsb.org (accessed on 3 September 2023)) (Entry ID: 6DO6, 3IFB, 6AQ1, 5HZ6, 1JJJ, 1O1V, 7E25, 7O60, 4A60, respectively) and AlphaFold (prediction) for FABP12 (Entry ID: A6NFH5) [12,13]. (**b**) The amino acid sequences of human FABP isoforms (FABP1-9, and FABP12) are illustrated using the multiple-sequence alignment tool, Clustal Omega. The asterisk (*) indicates the conserved amino acids. These amino acid sequences are mature protein sequences and do not include signal peptides in them. Amino acid sequences were obtained from the National Center for Biology Information (NCBI) website (www.ncbi.nlm.nih.gov/ (accessed on 3 September 2023)): FABP1 (NCBI Reference Sequence: NP_001434.1), FABP2 (NCBI Reference Sequence: NP_000125.2), FABP3 (GenBank: CAG33148.1), FABP4 (GenBank: CAG33184.1), FABP5 (NCBI Reference Sequence: NP_001435.1), FABP6 (GenBank: AAH22489.1), FABP7 (GenBank: CAG33338.1), FABP8 (GenBank: CAG46538.1), FABP9 (NCBI Reference Sequence: NP_001073995.1), and FABP12 (NCBI Reference Sequence: NP_001098751.1).

In humans, there are ten isoforms of fatty-acid-binding proteins (FABPs), each with a low molecular weight of approximately 14–15 kDa [5]. The classification emphasizes the organ or tissue where the isoform is most expressed [6,7]. However, no FABP is exclusively expressed in only one tissue [1]. These ten isoforms consist of FABP1/L-FABP (liver), FABP2/I-FABP (intestine), FABP3/H-FABP (heart/cardiovascular), FABP4/A-FABP (adipose), FABP5/E-FABP (epidermis), FABP6/IL-FABP (ileal), FABP7/B-FABP (brain), FABP8/M-FABP (myelin), FABP9/T-FABP (testis), and FABP12/R-FABP (retinal), and each protein isoform has a slightly different 3D structure, as represented in Figure 1a, reflecting their amino acid sequences [8]. Although there are variations in the amino acid sequences, the 3D structure of each isoform is highly conserved. This is because the sequence identity and similarity between isoforms are quite high, which indicates that there are no drastic changes in properties caused by amino acid variations in each isoform, such as polarity and charge, which can significantly affect the folding of the protein formed. However, small structural differences in the proteins result in the different ability of each isoform to bind various ligands [1]. For example, L-FABP has the capacity to bind to a broader range of ligands that span from lysophospholipids to heme [9], while B-FABP has the ability to bind docosahexaenoic acid and is said to specifically bind to other long-chain fatty acids [10]; thus, the capacity of each isoform to bind ligands is closely related to their role in lipid metabolism in the tissues where they are expressed. In general, the 3D structure of FABP consists of a 10-stranded antiparallel β-barrel structure, which is composed of two five-stranded β-sheets that are orthogonal [11]. The β-barrel contains a cavity that acts as a binding site for hydrophobic ligands. Small differences in the amino acid sequences result in variations in the amino acids that make up the cavity ligand-binding site formed in each FABP isoform. The amino acid sequences of each FABP isoform can be seen in Figure 1b. Although there are differences in the sequence of each isoform, each isoform has five conserved amino acid sites that indicate their family relationship as FABPs.

Fatty-acid-binding proteins (FABPs) are a class of polygenic proteins that have been linked to numerous metabolic disorders, including atherosclerosis, diabetes, and obesity [1]. Furthermore, intracellular FABPs have a function in the maintenance of lipid droplets, which is significant in the development of cardiovascular disease by controlling fatty acid absorption [6]. The expression of FABPs is typically increased in circumstances of inflammation and ischemia. Furthermore, it serves a crucial function in shielding cells from the harmful effects of fatty acids [14].

Due to their wide expression and role as a fatty acid transporter, FABPs are said to be involved in various metabolic diseases. These findings led to the proposal of FABPs as potential candidates as biomarkers for various diseases of tissues and organs that express it. In this review, we will discuss these findings on the role of several FABPs that have been reported to have the potential and development as disease biomarkers as one of the considerations for developing them as therapeutic agents and/or diagnostic methods.

## 2. Method

This literature study was written based on findings from previous studies by collecting, categorizing, selecting, and reviewing research results according to relevant topics. The keywords used in searching and collecting literature are fatty-acid-binding protein/FABP, biomarker, diagnosis, diseases related to FABP, and overexpression/high expression/high level of FABP. The reviewed literature was collected through various online scientific journals and book portals.

## 3. FABP1/L-FABP

L-FABP is extensively expressed in the liver (hepatocyte cytoplasm) and has been found in other tissues, like kidneys (renal tubules and proximal tubules), intestines (enterocytes), lungs, stomach, and pancreas [1,15,16]. L-FABP is believed to be capable of binding two ligands concurrently via two separate binding sites (low and high affinity); it can also bind molecules that have the potential to be cytotoxic, such as heme groups, apart from fatty acids [1,15].

In a study of infants with necrotizing enterocolitis (NEC), Benkoe et al. (2014) discovered a substantial rise in serum levels of I-FABP, L-FABP, and IL-8 (interleukin-8). Through their research, there were 15 infants with NEC involved and they were grouped into two groups based on different treatments, namely, medical NEC and surgical NEC. They measured the levels of I-FABP and L-FABP using ELISA and compared them with the level of IL-8 as a proinflammatory chemokine. The study has shown that all three have the ability to detect NEC, indicating that they could be potential biomarkers for the disease. Although the highest ratio between infants in the control group and infants with NEC was shown by IL-8 compared to both FABPs, they concluded that IL-8 is considered to be superior to I-FABP, its diagnostic ability is similar to L-FABP, and IL-8 is more significant as a biomarker for NEC [5]. However, the population tested in this study is still considered insufficiently representative, and further research with a larger population is needed to support the results of this study.

Furthermore, in multiple investigations, L-FABP has been investigated as a potential biomarker for liver and kidney injury [17,18,19,20,21]. It is thought to be a potential biomarker for acute kidney injury (AKI) [22,23]. Naruse et al. (2018) conducted a study on a population of 1273, which was divided into 224 patients with AKI and 1049 non-AKI. They collected urine and blood samples and measured the level of L-FABP using a latex-enhanced immunoturbidimetric assay. The results of this study reported that the combination of L-FABP and NT-proBNP measurements as the early detection of AKI in patients provides better prediction. However, this study has limitations because the treatments in this study were not controlled or randomized, so the administration of different treatment strategies to patients may affect the evaluation, and the observation of its effects on the progression of AKI in patients becomes difficult. Nonetheless, multivariate logistic analyses performed with a variety of factors showed that this study has good significance and validity for AKI [24]. It has also been observed that L-FABP can distinguish between AKI and acute-on-chronic liver failure (ACLF) in cirrhotic patients. A study by Graupera et al. (2017) revealed that L-FABP has the potential to be applied as a biomarker for detecting liver injury caused by cirrhosis, as L-FABP levels were observed to be greater in individuals with cirrhosis compared to healthy subjects [25]. Through the research conducted by Belcher et al. (2014), testing was performed on 188 patients with cirrhosis and AKI by measuring L-FABP and other biomarker levels. The measurement of L-FABP in the study used anti-human L-FABP antibodies that have been developed and the ELISA method. The study concluded that L-FABP, along with three other biomarkers (NGAL/Neutrophil gelatinase-associated lipocalin, IL-18/Interleukin-18, and albumin), has a good and efficient ability to differentiate patients with progressive AKI and cirrhosis. Although this study has comprehensively observed each biomarker, it cannot standardize the treatments performed by patients, so the effect of treatment response on biomarker levels cannot be ascertained and assessed [26]. A similar study by Juanola et al. (2021) also indicates the potential of L-FABP as a biomarker of complications for patients with decompensated cirrhosis. This study was based on 444 patients with decompensated cirrhosis, and L-FABP measurements were performed using the Human L-FABP ELISA kit based on plasma and urine samples. The results of the study showed that L-FABP can predict the mortality rate and detect ACLF in patients with decompensated cirrhosis. However, there is still a need for further studies regarding the contribution of L-FABP in the development of ACLF which was not obtained through this study [27]. Furthermore, some researchers have suggested that L-FABP could be used as a biomarker for graft failure in kidney transplant recipients [16,28].

L-FABP was found to be useful in detecting hepatocyte injury in individuals having a liver resection, according to Van De Poll et al. (2007). Through this research, they studied ten patients undergoing liver resection and took arterial blood samples before and after the operation to measure the level of L-FABP. The number of test populations in the study is still too small to represent and strengthen the test results, but, through this study, a hypothesis was obtained regarding the participation of L-FABP in inflammation in the intestines and liver; thus, they concluded that L-FABP is referred to as an important biomarker in measuring patient plasma because of its role in systemic inflammation [29]. Based on the considerable increase in blood serum L-FABP levels in patients with acute hepatocellular damage, Pelsers et al. (2002) state that L-FABP can be used as a biomarker for diagnosing acute hepatocellular damage and sensitive liver transplant patients. The study was conducted on 21 liver transplant recipients; the criteria of the subjects were patients who had experienced acute hepatocellular rejection several times during the post-transplantation recovery process at the hospital. The results showed that L-FABP was as good as, or even better than, a-GST for early detection and had a significantly high level in liver transplant patients with acute hepatocellular damage. Although the number of patients studied was not sufficient to represent the research results and further research is needed on this finding, this study has been a good guide in the discovery of this hypothesis [30]. According to a study by Karvellas et al. (2017), serum L-FABP levels were measured in 198 patients with APAP-ALF using ELISA. It has been reported that serum L-FABP levels rise in patients with acetaminophen-induced acute liver failure (APAP-ALF). Because an increase in serum L-FABP levels is related to an increased risk of patient mortality, L-FABP is suspected of having the capacity to discriminate between survivors and non-survivors in patients with APAP-ALF. This study is comprehensive and able to present the role and contribution of L-FABP in APAP-ALF quite clearly, making it a strong foundation to support the research data [31].

A substantial rise in serum levels of L-FABP was found in chronic hepatitis C (CHC) patients with liver injury compared to CHC patients without liver injury. According to Akbal et al. (2013), L-FABP had an 80% sensitivity and 100% specificity in diagnosing liver injury in CHC patients. In this study, testing was performed on 42 patients, consisting of 20 healthy controls and 22 patients with CHC. The results showed a strong correlation between the level of L-FABP and the level of hepatitis C virus (HCV) RNA, alanine aminotransferase, and hepatic inflammation. However, further research on these findings is needed, especially in the case of normal alanine aminotransferase and higher HCV RNA levels [32].

Other research has suggested that L-FABP, together with I-FABP, could be used as a marker to detect severe abdominal injuries in patients who have suffered numerous traumas. The study discovered an increase in both FABP levels in patients with multiple traumas compared to healthy subjects, which is associated with the severity of abdominal injury in patients. In this study, L-FABP and I-FABP levels were measured in blood samples from 102 patients with multiple traumas and 30 healthy subjects using ELISA. Although the results of the study showed good significance, future clinical research with a larger test population is needed to validate the potential of L-FABP and I-FABP as detectors of severe abdominal trauma in patients [33].

## 4. FABP2/I-FABP

I-FABP is expressed in stomach and intestinal epithelial cells and can be quantified in the form of plasma (I-FABPp) and urine (I-FABPu) [1,34]. I-FABP co-expresses with L-FABP and Il-FABP in the small intestine but is distributed differently. L-FABP is predominantly expressed in the proximal region, while Il-FABP is predominantly expressed in the distal region. I-FABP, on the other hand, is distributed throughout the intestine but is most abundant in the distal part [1]. I-FABP also plays an important role in the uptake of dietary fatty acids in enterocytes [35].

As we mentioned before, in a study conducted by Benkoe et al. (2014), I-FABP was revealed as one of the relevant biomarkers for identifying NEC, and, although I-FABP has the lowest value among the three, they found the potential of I-FABP to differentiate between infants with surgical NEC and medical NEC to be in accordance with the increased level of I-FABP, but this increase did not reach statistical significance [5]. However, several additional papers have also suggested I-FABP’s potential as a biomarker for NEC. In a study by Schurink et al. (2014), plasma I-FABP and urine I-FABP levels were evaluated in infants suspected of having NEC. In this study, measurements and analysis of I-FABP levels were conducted on urine and plasma samples from 37 infants with NEC using the ELISA method. The results of the study showed that both plasma I-FABP and urine I-FABP are significantly correlated with NEC and have the potential to develop a diagnostic method for infants with suspected NEC through the measurement of I-FABP. The study reports a strong correlation between urine I-FABP and other conventional NEC biomarkers such as IL-6, WBC, and lactate. They hypothesized that NEC occurs due to ischemia of the upper villi, which leads to enterocyte damage and the subsequent release of I-FABP into the bloodstream. This is what connects I-FABP with lactate in the early stages of NEC. The test population size is still considered insufficient to represent and explain the participation of I-FABP in NEC onset, so further research is needed regarding the potential of I-FABP as a biomarker for NEC [34]. These findings are supported by Abdel Haie et al. (2017), which has a similar report about this hypothesis. This study was conducted on 160 preterm neonates who were less than 35 weeks old and weighed less than 2000 g. After the observation period, 18 of them developed NEC (group 1), while the remaining 10 were used as healthy controls (group 2). The I-FABP levels were measured in both groups using an ELISA kit. The results of the study showed an increase in I-FABP levels in neonates with NEC compared to healthy neonates. This increase in I-FABP levels was found to be correlated with the severity of NEC (stage 1, stage 2, and stage 3). Therefore, the researchers hypothesized that measuring I-FABP could be an early detection method for neonates with NEC. The statistical analysis also indicated that serum I-FABP has high specificity and sensitivity as a biomarker. However, the number of subjects tested was considered relatively low, and further research with a larger sample size focusing on the ability of I-FABP to differentiate the severity levels in neonates with NEC is needed to confirm the findings of this study [36,37].

Another study by El-Abd Ahmed et al. (2020) also supports the finding. In this study, plasma and urine I-FABP levels were analyzed by using ELISA on 55 neonates with NEC and 23 healthy neonates as controls. Through the study, it was reported that there is a different role between plasma I-FABP and urine I-FABP. Plasma I-FABP has been discovered as a potential detector of surgical NEC for early diagnosis, whereas urine I-FABP has been discovered as a marker that differentiates between Bell’s stage II and Bell’s stage III in NEC cases. The statistical analysis also mentioned that urine I-FABP has better specificity compared to plasma I-FABP, while both have the same sensitivity. This study provides deeper findings regarding the potential of I-FABP as a biomarker for NEC by conducting testing and comparing plasma and urine I-FABP in differentiating NEC stages [37]. The role of I-FABP in the pathogenicity of NEC has not been widely discussed. This could be a suggestion for further research so that the hypothesis regarding the potential of I-FABP as a biomarker for NEC can be supported by more comprehensive knowledge.

I-FABP has also been reported to detect intestinal ischemia based on several works of literature. Niewold et al. (2004) discovered that I-FABP levels rose in pigs with intestinal ischemia [14]. In their investigation of the involvement of I-FABP and D-lactate in intestinal ischemia, Shi et al. (2015) discovered a significant rise of the concentration levels of I-FABP and D-lactate. A total of 272 patients with severe abdominal pain symptoms had their blood samples taken to measure the levels of I-FABP and D-lactate using an ELISA kit. Among these patients, there were 39 patients diagnosed with intestinal ischemia and 233 patients who did not have ischemia. When enterocytes are damaged, I-FABP, a low-molecular-weight intracellular protein, is found exclusively in the small intestine and, consequently, released and circulated, resulting in a rise in I-FABP levels. The sensitivity and specificity of I-FABP have been reported to be 90.0% and 86.7%, respectively, for the detection of acute intestinal ischemia. Although I-FABP has the highest sensitivity compared to other biomarkers, its specificity is still lower than that of D-lactate, which has the highest value among other biomarkers. Therefore, the application of I-FABP as a single biomarker may not be recommended and combining it with D-lactate may provide better predictive results [38]. Another study has found comparable results in terms of I-FABP’s potential as a biomarker for acute intestinal ischemia, particularly in patients with small bowel ischemia. The analysis and measurement of I-FABP levels in 40 patients with symptoms of acute intestinal ischemia using an ELISA kit showed that I-FABP has a sensitivity and specificity of 95.7% and 88%, respectively. This study concluded that I-FABP is a novel biomarker that is fast, sensitive, specific, and cost-effective for intestinal/bowel ischemia. However, this study did not perform a comparative analysis with other biomarkers, so the application of I-FABP as a single biomarker for early detection and diagnosis cannot be confirmed based solely on these findings [39]. Camara-Lemarroy et al. (2021) similarly reported high serum I-FABP and D-lactate concentrations in individuals with acute ischemic stroke (AIS), confirming I-FABP’s potential as a promising biomarker. In this study, 61 patients with acute ischemic stroke were tested for I-FABP levels using an ELISA kit. Among them, 20 patients had small-vessel disease, 20 patients had cardioembolic stroke, 21 patients had atherosclerosis, and 20 healthy patients were included as controls. The study considered the factors causing acute ischemic stroke as testing variations and hypothesized the possibility of intestinal mucosa/barrier damage in patients with acute ischemic stroke. However, this study has not been able to directly prove the occurrence of intestinal injury in patients with acute ischemic stroke. Therefore, further research with a larger number of test subjects is needed to validate these findings and investigate the mechanisms and correlation of I-FABP with acute ischemic stroke clinically [40].

A study by Vermeulen et al. (2012) also discovered that I-FABP has the potential to be applied as an early detection biomarker for intestinal necrosis in post-aortic surgery patients by measuring the circulating level of I-FABP. The testing in this study involved 96 patients who had undergone aortic surgery, classified into three groups: OR-TAA(A) (open repair of a thoracic or thoracoabdominal aortic aneurysm), AAA (open infrarenal or juxtarenal abdominal aortic aneurysm repair), and EVAR (endovascular aneurysm repair) with 55, 25, and 16 patients, respectively. The I-FABP level measurement was performed using an ELISA kit. The measurement results showed the potential of I-FABP in detecting intestinal injury in patients with open aortic repair, and no intestinal injury was detected in patients with EVAR. Further research with a larger population of patients is needed to test the ability of I-FABP to detect lethal postoperative intestinal injury to confirm the hypothesis in this study [41]. Another finding from Relja et al. (2010) also reported that I-FABP has the potential to be used as a marker for severe abdominal injury along with L-FABP, as previously mentioned [33].

## 5. FABP3/H-FABP

H-FABP is broadly expressed in numerous organs such as the brain, lungs, testis, and renal cortex. However, it is mainly expressed in skeletal muscle and the heart [1,6,42]. When there is muscle injury, H-FABP is involved in the transfer and metabolism of fatty acids and will be released into the circulation at the site of injury [6,42,43]. H-FABP is a small protein that regulates myocardial fatty acid metabolism, which is expressed in cardiomyocytes. Its expression concentration increases in acute ischemic stroke and acute myocardial injury (MI) conditions [44]. Additionally, H-FABP is exclusively expressed in neurons. While H-FABP is hardly noticeable in the brain during embryonic development, its expression increases gradually from birth until it reaches adulthood [45].

H-FABP has been associated with patients with chest pain, as mentioned in several studies. Bivona et al. (2018) conducted research on H-FABP and found that it can be used as a novel marker for acute coronary syndrome (ACS). A study found that individuals with acute coronary syndrome (ACS) had considerably greater levels of H-FABP expression than healthy controls. According to the study, H-FABP has the capacity to predict the risk of major adverse cardiovascular events (MACEs) in ACS patients [44]. Reddy et al. (2016) reported similar findings; through their study involving 88 patients with ACS and non-ACS, H-FABP had better sensitivity while its specificity was lower than hs-TropT. Therefore, the combination of hs-TropT and H-FABP can provide a more precise prediction for ACS than their separate use [46].

H-FABP is also referred to as a novel marker for the early detection of myocardial infarction. Collinson et al. (2014) compared the diagnostic accuracy between H-FABP and troponin for patients with myocardial infarction and they discovered that H-FABP can be used as a marker for diagnosing myocardial infarction. However, cardiac troponin is considered a better marker in terms of sensitivity. Combining both markers is believed to optimize the diagnostic method for myocardial infarction for patients with symptoms, such as chest pain. This study involved 850 randomized patients, and, after testing, 68 of them were diagnosed with myocardial infarction. The test results reported that the detection ability of H-FABP is lower than troponin, but the combination of both can increase diagnostic sensitivity. Therefore, H-FABP is not recommended as a single biomarker because its specificity is lower than that of troponin. Nevertheless, this study has reported comprehensive findings on the potential of H-FABP as a myocardial infarction biomarker, and the test subject scale is sufficient to represent the reported findings [47]. This statement is supported by Vupputuri et al. (2015), which reported that H-FABP is a biomarker with high sensitivity and has the potential to be developed for the acute myocardial injury (AMI) early detection biomarker and can even be used for patients with unstable angina. This study involved 54 patients with acute chest pain by measuring the level of H-FABP and other biomarkers using a latex-enhanced immunoturbidimetric assay. The test results showed that H-FABP has a greater sensitivity compared to other biomarkers, making it a better choice for early detection. Therefore, combining H-FABP as the early detection biomarker and cardiac troponin as the late detection marker can potentially develop an optimal method for diagnosing myocardial injury [48].

Pyati et al. (2015) stated that H-FABP can be applied as the early detector of myocardial injury and is even considered a better early detector for acute myocardial injury due to its sensitivity and specificity in patients with chest pain within 3–6 h of symptom onset compared to CK-MB and myoglobin. This study involved 40 patients with AMI and 40 healthy controls, with CK-MB, myoglobin, and H-FABP levels measured using immunoturbidimetric, immunoinhibition, and chemiluminescence immunoassay methods, respectively. The test results showed that H-FABP had the highest PPV (positive predictive value), NPV (negative predictive value), specificity, and sensitivity compared to the other two biomarkers in detecting patients with AMI. Although this study has provided significant data and comprehensive methods, the number of patients tested is considered insufficient. Further testing with a larger sample size will be able to confirm the findings of this study [49]. Agnello et al. (2017) also reported that H-FABP can detect chest pain in patients more sensitively within one hour of symptom onset compared to cardiac troponin. This study involved 28 patients with AMI and 28 patients with non-AMI who experienced chest pain within an hour of the onset of pain. The measurement of H-FABP and hs-TnI (cardiac troponin) in the patient’s blood samples was performed using an immunoturbidimetric assay. However, the specificity of H-FABP is still lower than hs-TnI. Therefore, the application of H-FABP as a single marker is still considered inadequate. In addition, this study used a very small sample size, so the validity of these findings cannot be confirmed until further testing is conducted with a more sufficient population size. Nevertheless, the ability of H-FABP to detect AMI in patients with chest pain within an hour is a great potential that can prevent fatal conditions from occurring in patients and provide an opportunity for faster treatment if the development of H-FABP as a biomarker can be further studied [50]. In addition, H-FABP is also mentioned as a novel biomarker that has the ability to predict the diagnosis and prognosis of peripheral arterial disease (PAD), by measuring H-FABP and other biomarkers in 1200 patients with PAD and non-PAD using ELISA. The test results reported that H-FABP and N-terminal pro-B-type natriuretic peptide have the best predictive ability among other biomarkers. H-FABP is strongly associated with the severity of PAD and shows the strongest correlation with PAD and CLI (critical limb ischemia) with sensitivity and specificity values of 91% and 100%, respectively [51]. This is due to its high expression in PAD patients with a history of coronary arterial disease (CAD) or diabetes mellitus, as H-FABP is released and circulates when skeletal muscle injury occurs [52].

In addition to its potential as a biomarker for myocardial injury and peripheral arterial disease, H-FABP is also being studied as a novel biomarker for Alzheimer’s disease (AD). Guo et al. (2013) found that combining H-FABP and VEGF in cerebrospinal fluid (CSF) with previously discovered markers, namely, Aβ_1–42_, tTau, and pTau_181_, is an optimal early diagnostic method for AD. The study involved 149 patients with mild cognitive impairment (MCI), 69 patients with AD dementia, and 92 controls. The test results showed that H-FABP and VEGF (vascular endothelial growth factor) were able to detect AD dementia, and H-FABP was said to be capable of predicting the progression of MCI into AD in patients, but the specificity and sensitivity of FABP were lower than those of VEGF. They reported that optimal detection was achieved when H-FABP and VEGF were combined with the three established markers, resulting in an increase in specificity and sensitivity to 86% and 83%, respectively. This study did not analyze the relationship between H-FABP and VEGF and cardiovascular risk in AD patients. Further research to analyze the impact of cardiovascular risk on AD patients may be needed to better understand the correlation between H-FABP and AD pathology [53]. Sepe et al. (2018) also reported that H-FABP is related to the accumulation pathway of α-Syn and the deregulation of dopaminergic pathways in synucleinopathy, and found that the expression level of H-FABP is associated with α-Syn aggregation in synucleinopathy [54]. However, this mechanism is still not clearly understood and requires further research.

## 6. FABP4/A-FABP

A-FABP is generally found in matured adipocytes and macrophages. It has been reported to play a part in inflammatory and metabolic processes and is involved in the occurrence of insulin resistance, diabetes, and atherosclerosis [55]. In some physiological and pathological tissues, A-FABP is predominantly found in microvascular endothelial cells (ECs). Endothelial A-FABP promotes cell proliferation, survival, and migration by inducing a pro-angiogenic role [56]. A-FABP also affects the activity of ECs by controlling the expression of the activator genes of ECs, in particular, eNOS (endothelial nitric oxide synthase) and ICAM-1 (intercellular adhesion molecule 1) [57].

Hotamisligil et al. (2015) mentioned that reducing the expression level of A-FABP and E-FABP can enhance glucose homeostasis and lower the risk of atherosclerosis in mice; circulating A-FABP is said to have an important hormonal function in the metabolic system [3]. A-FABP is also implicated in the development of breast cancer, particularly for those with obesity, due to its high expression [58]. This hypothesis is supported by a study by Hao et al. (2018) which reported the potential of A-FABP as a novel functional biomarker for protumor TAM (tumor-associated macrophage) and its potential as an immunotherapy target for patients. This study measured and analyzed the expression level of A-FABP in wild-type mice (A-FABP+/+) and A-FABP knockout mice (A-FABP−/−) using Western blotting and miRNA microarray methods, as well as observations using human samples of normal/benign breast tissues and malignant breast tissues using a confocal microscope. The study reported the high expression of A-FABP in a subset of macrophages (CD11b^+^F4/80^+^MHCII^−^Ly6C^−^) that directly contribute to tumor cell growth. A-FABP plays a role in mediating tumor development through the miR-29b/IL6/STAT3 cascade. When a subset of macrophages that contain A-FABP accumulates in the tumor stroma, tumor development through this pathway also increases [59]. Therefore, there is a hypothesis that A-FABP could be a potential biomarker for detecting obesity-based breast cancer. In addition to breast cancer, Zhang et al. (2019) reported that A-FABP, along with Il-FABP, can be developed as a potential biomarker for detecting colorectal cancer because they are expressed at high levels in colorectal cancer patients compared to healthy individuals. In this study, A-FABP and Il-FABP serum levels were measured in 100 patients (38 with colon cancer and 62 with rectal cancer) who had been diagnosed with colorectal cancer without surgery, radiotherapy, or chemotherapy using the ELISA method before and after 2 weeks of radical resection of colorectal cancer. Immunohistochemistry and Western blotting were then performed to observe the expression of A-FABP and Il-FABP in colorectal tumor tissues and adjacent tissues. The results showed that A-FABP and Il-FABP serum levels were significantly higher in colorectal cancer patients before surgery than two weeks after surgery, where there was a drastic decrease. Multivariate logistic regression analysis also showed an increased risk of colorectal cancer in patients with A-FABP and Il-FABP serum levels independently. This suggests that A-FABP and Il-FABP serum levels may have a strong correlation with the development of colorectal cancer. However, they could not find a correlation between A-FABP and Il-FABP serum levels and clinicopathologic features of colorectal cancer in this study. Therefore, further research is needed to validate these findings with a more adequate test population and deeper analysis related to the role of A-FABP and Il-FABP in the progression of colorectal cancer [60].

## 7. FABP5/E-FABP

E-FABP is mainly expressed in skin epidermis cells and is also present in various organs such as the tongue, adipose tissue, macrophages, dendritic cells, brain, mammary gland, liver, kidney, lung, and testis [1]. E-FABP has a primary macrovascular expression pattern in endothelial cells (EC) [61]. Despite having a high amino acid sequence homology (55%) with A-FABP, its function in ECs is not well understood. However, studies have reported that both A-FABP and E-FABP play a role in the absorption of fatty acids in ECs from the heart and skeletal muscle [61].

As previously mentioned, a publication by Hotamisligil et al. (2015) found that inhibiting the expression of E-FABP, along with A-FABP, can reduce the risk of atherosclerosis and improve glucose homeostasis in mice. This is related to the significance of FABPs as mediators in the processes of inflammation and metabolism [3].

Another study by Ohata et al. (2017) identified that an increase in the expression level of E-FABP in hepatocellular carcinoma (HCC) tissue is associated with HCC progression. Therefore, E-FABP is considered a potential biomarker for detecting HCC and predicting the risk of HCC progression. In this study, E-FABP levels in liver tissues were measured from 243 patients who had undergone surgical resection for HCC using immunohistochemistry, Western blot, and xenograft tumor growth observation. This study reported the overexpression of E-FABP in human HCC tissues, and this overexpression was associated with tumor size, poor cell differentiation, vascular invasion, and distant metastasis. Based on the results of a multivariate analysis, E-FABP overexpression is one of the strong risk factors associated with survival and recurrence. In vivo testing in mice showed that E-FABP overexpression affects tumor growth, as well as proliferation, invasion, and migration in vitro testing. These findings support the hypothesis that E-FABP plays a role in tumor development, metastasis, and invasion in HCC patients. However, further testing is still needed to confirm the metabolic role of E-FABP in HCC pathogenicity [62]. This is supported by Liu et al. (2020); in this study, 138 patients who had been diagnosed with HCC were tested by taking tissue samples of HCC and the adjacent normal liver from each patient. This study suggests that E-FABP can be applied as a potential marker for diagnosing and predicting the prognosis of HCC. Although the test population was inadequate, this study was able to explain the hypothesized correlation between E-FABP levels and the prognosis of HCC. Therefore, further research with a larger test population and more in-depth clinical testing will be needed [63].

E-FABP has been widely mentioned to play a role in the development of various cancers, especially those of the colon, prostate, and breast. In breast cancer, E-FABP is said to be involved in HER2 tumorigenesis, which is one of the epidermal growth factor receptors [64]. Studies have shown that E-FABP is pro-tumorigenic in patients with triple-negative breast cancer (TNBC) by playing a role in retinoic acid signaling [65]. This is supported by Powell et al. (2015), who reported that E-FABP is involved in metastasis and tumor growth by comparing the size and development of TNBC cells in wild-type mice with E-FABP knockout mice [66]. Another study by Levi et al. (2015) also reported interesting findings regarding the relationship between E-FABP and long-chain fatty acids (LCFA), which consists of two types: saturated (SLCFA) and unsaturated (ULCFA). E-FABP is described as an important mediator of the peroxisome proliferator-activated receptors (PPARβ/δ) signaling response in both types of LCFA. SLCFA is reported to block E-FABP and inhibit the activation of PPARβ/δ, while ULCFA is transported by E-FABP to PPARβ/δ and activates it, suggesting the development of E-FABP inhibitors as a potential therapeutic method [67]. Research by Ju et al. (2018) also supports the same hypothesis, as the study reported that 4-Amino-2-trifluoromethyl-phenyl retinate (ATPR) is an E-FABP inhibitor, and the inhibitory effect shows a decrease in breast cancer proliferation by inducing cell differentiation. In addition, E-FABP expression was reported to be increased in the tested breast cancer tissues, but its level was low in benign fibroma breast tissues, indicating that an increase in E-FABP level may be associated with tumor development [68].

E-FABP serves an important role in the tumor growth and metastasis of prostate cancer, as reported in several publications. Studies have shown that E-FABP is not present in a normal prostate, but it is highly expressed in advanced metastatic prostate cancer [69,70,71,72,73]. This is due to changes in fatty acid metabolism in cancer cells, especially prostate cancer, due to the high need of cells for fatty acids during the process of cancer cell proliferation as a source of energy, the formation of membrane components, and the production of signaling molecules [74], so several reports mention that limiting fatty acid intake can inhibit the development and proliferation of cancer cells [74,75,76]. Consistent with these reports, differences in E-FABP expression levels were found in weakly metastatic cell lines and aggressive metastatic cell lines, which were low or even absent and high, respectively [77,78,79]. High levels of E-FABP expression are said to promote metastasis and tumor growth, so pharmacological or genetic inhibition of E-FABP can suppress prostate cancer metastasis [80]. The activation of PPARγ and estrogen-related receptor α mediates the pro-metastatic role of E-FABP [81]. In a study by Carbonetti et al. (2019), fatty acid synthase (FASN) and monoacylglycerol lipase (MAGL) were used as prototypes to demonstrate that the products of FASN, MAGL, and possibly other lipid-metabolizing enzymes depend on E-FABP5 for PPARγ activation in the nucleus. FASN and MAGL are reported to play a role in increasing prostate cancer metastasis through in vitro and in vivo analysis and are dependent on E-FABP because of their role as cytosolic lipid transport proteins to nuclear receptors that are important for prostate cancer metastasis, along with the increase in E-FABP levels and amino acid metabolism in the process of prostate cancer metastasis [82]. E-FABP has been identified as a key player in the signal transduction pathway of castration-resistant prostate cancer (CRPC), which is crucial in converting cancer cells from being androgen-dependent into androgen-independent. In this signaling, the levels of E-FABP increase along with the increasing malignancy of cancer cells [79]. Both intracellular and extracellular fatty acids are present in high amounts and are then delivered to the nucleus of cancer cells by E-FABP as a signaling molecule to activate PPARγ. PPARγ, once activated, will express its target genes, resulting in the increased expansion and aggressiveness of cancer cells due to overgrowth caused by low apoptosis and high angiogenesis [77]. Therefore, E-FABP is said to be a potential therapeutic target for prostate cancer through inhibitory mechanisms.

In addition to the PPARγ signaling pathway, interesting findings by Senga at al. (2018) in their study hypothesized the role of E-FABP in the process of prostate cancer cell proliferation and metastasis through another pathway, namely, ERRα. Through their research, they used the PPARβ/δ agonist GW0742 to test its effect on the E-FABP-PPARβ/δ signaling pathway. It was reported that the increased expression of metabolic genes (3-phosphoinositide-dependent protein kinase-1, adipose differentiation-related protein, and integrin-linked kinase), which play a role in phosphorylating AKT (protein kinase B) to activate survival signaling, induced by GW0742 did not affect E-FABP levels. Likewise, neither the overexpression nor knockdown of E-FABP levels affected the expression of metabolic genes in both GW0742 and non-GW0742 treated cells. This suggests that E-FABP is not related to or independent of the PPARβ/δ signaling pathway in prostate cancer cells and its role in proliferation and metastasis is through other pathways. Other findings in the study reported that E-FABP modulates the expression of other metabolic genes (ATP synthase subunit beta, long-chain 3-hydroxyacyl-CoA dehydrogenase, aconitase 2, fumarate hydratase, and mitofusin-2) and energy metabolism through the ERRα pathway by acting as a transcriptional factor for these metabolic genes in the nucleus. This is supported by the finding of specific interactions between E-FABP with ERRα and PGC-1β, which are co-activators for ERRα and PPARs and regulators of energy metabolism [81].

Through these findings, it can be said that the role of E-FABP on cancer cell proliferation and metastasis is quite significant and needs to be further developed in both therapeutic and diagnostic approaches. Its high expression level in breast and prostate cancer cells can be one of the potential detection indicators for diagnosis. Several studies have proposed E-FABP as a biomarker for the detection of both types of cancer. Another publication reported an overexpression of E-FABP in prostate cancer and is suspected to be associated with poor prognosis and low survival rates. It was found, through in vitro observation, that reducing the expression of E-FABP through the knockdown or knockout of E-FABP can reduce prostate cancer proliferation and invasiveness, as well as inhibit metastasis and tumor growth in vivo [83]. In addition to prostate and breast cancer, another study has found that E-FABP might correlate with cervical cancer by analyzing 206 cervical squamous cell cancer samples using immunohistochemical testing; it also reported a significant rise in the E-FABP level, and E-FABP’s role in the development of cervical cancer is suspected. Silencing E-FABP led to a reduction in cell proliferation, migration, colony formation, and invasiveness in vitro, as well as a significant decrease in tumor growth in vivo. Therefore, E-FABP is referred to as a potential predictor for the prognosis and diagnosis of cervical cancer patients [84]. However, this hypothesis still needs further study to confirm the significance of the role of E-FABP in the metastasis and carcinogenesis of cervical carcinoma and their correlation with the poor prognosis of cervical cancer.

## 8. FABP6/Il-FABP

Il-FABP, commonly known as gastrotropin, is most abundant in the ileum. It helps transport bile acids and has the ability to bind to bile acids stronger than fatty acids, which is why it is also called ileal-bile-acid-binding protein (IBABP) [85,86]. Il-FABP is also expressed in the small intestine’s distal area, where it works as a transporter for intercellular bile acid in ileal epithelial cells. This transporter plays a role in the catalysis mechanism, as well as cholesterol metabolism [60]. In a study by Zhang et al. (2019), as previously described, Il-FABP was mentioned as a potential biomarker for colorectal cancer, with higher levels of Il-FABP found in patients with colorectal cancer. However, this study is still limited to measuring patients after two weeks postoperatively, and there is a need to consider the prognosis of postoperative patients over a longer period of time. Therefore, the relationship between the potential of Il-FABP as a diagnostic and prognostic biomarker cannot be identified at this time. However, the combination of A-FABP and Il-FABP with CEA (serum carcinoembryonic) can provide more sensitive and accurate diagnostic capabilities for colorectal cancer [60].

## 9. FABP7/B-FABP

B-FABP is a protein that has a strong attraction to n-3 PUFA and is mainly found in astrocytes and oligodendrocytes. It is abundantly expressed in the embryonic brain, while its expression decreases in the adult brain. B-FABP is referred to as an essential protein for the growth and maintenance of neural stem cell progenitor and radial glial cells [1,87].

B-FABP expression levels have been found to be raised in various neurodegenerative diseases. It has been reported that high levels of B-FABP mRNA expression have been found in the postmortem brains of patients who are diagnosed with autistic spectrum disorder [88] and schizophrenia [89]. This is also supported by a study by Iwayama et al. (2010) that reported a relationship between B-FABP expression and schizophrenia and bipolar disorder. Through this study, an analysis was performed on 950 patients with schizophrenia and 950 control subjects, as well as 867 patients with bipolar disorder and 895 control subjects. In this study, resequencing analysis and single nucleotide polymorphism (SNP) selection and genotyping were performed on B-FABP, E-FABP, and L-FABP from each subject of the study. The results showed a relationship between B-FABP and bipolar disorder with the discovery of three SNPs that showed a strong correlation with bipolar disorder, supported by an empirical relationship between haplotypes of the B-FABP gene and bipolar disorder even after several correction tests were performed. This study has provided comprehensive and in-depth data on the relationship between the B-FABP gene and schizophrenia and bipolar disorder. However, further analysis of the unknown role of regulatory elements on the B-FABP gene in mental disorders is considered necessary to confirm the findings of this study [90]. In another study, Teunissen et al. (2011) found a rise in B-FABP levels in the serum of individuals with Alzheimer’s disease (AD), Parkinson’s disease (PD), and other neurological disorders related to dementia compared to non-dementia patients, indicating that B-FABP has potential as a biomarker for these neurodegenerative diseases. In this study, the research subjects consisted of 31 AD patients, 43 PD patients, 42 OCD patients (other cognitive disorders), and 52 control subjects. The level of B-FABP serum was measured using the ELISA method and immunohistochemistry using a B-FABP-specific polyclonal antibody developed by the researchers themselves. Through their research findings, they hypothesized that the presence of B-FABP in astrocytes and the increased level of serum B-FABP in 29% of AD patients may be related to the role of B-FABP in the occurrence of AD. Although the results of the study showed the potential of B-FABP as a biomarker, its use as a single biomarker for diagnosis is not recommended due to the low percentage of cases with increased B-FABP in patients. The number of research subjects is also relatively low, and further research is needed with a more adequate test population and the use of more sensitive and accurate testing by developing other more specific antibodies such as monoclonal antibodies or antibody fragments specific to B-FABP [91]. However, more research is required to better understand B-FABP’s role in the development of these disorders.

## 10. FABP9/T-FABP

T-FABP is a protein that binds to fatty acids and is found in large quantities in the testis. It is responsible for maintaining the quality of sperm. In patients with prostate cancer, it has been thought to be influenced by the overexpression of T-FABP, which is believed to be a significant factor in its progression and development [92]. T-FABP was discovered to be overexpressed in prostate cancer tissue in a study conducted by Al-Fayi et al. (2016). In this study, testing was performed on benign and malignant prostate cell lines by measuring the level of T-FABP mRNA. An increase in the expression level of T-FABP was found in all malignant prostate cell lines compared to benign cell lines. This measurement was performed using the Western blotting method and immunohistochemistry staining based on the interaction of anti-T-FABP antibody with the expressed T-FABP. The measurement results showed a correlation between the increase in T-FABP levels and the decrease in patient survival rates. The increase in T-FABP is also said to be correlated with an increase in Gleason scores (GSs) and the androgen receptor index (AR). Through this testing, it was also reported that the decrease in T-FABP level can inhibit the invasiveness of highly malignant PC3-M cells. The study revealed that T-FABP could be used as a possible biomarker for diagnosing and predicting the development of prostate cancer [92]. However, there have not been many studies that have further explored this potential, so the role of T-FABP in the development of prostate cancer has not been fully understood. This study can be a suggestion for future researchers to clinically confirm these findings in patients with prostate cancer and investigate its role in cancer cells, which may open up new hypotheses about its potential as a therapeutic agent.

## 11. Discussion

**Figure 2:**
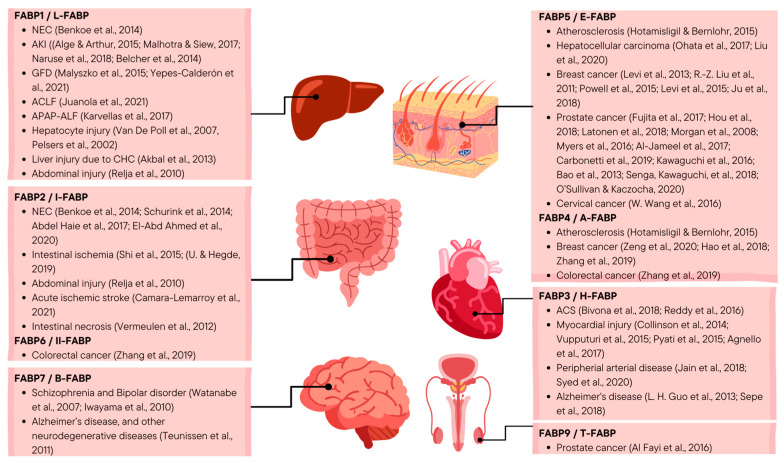
Summary of the potential FABPs as a disease biomarker (self-made figure, designed using Canva) [3,5,16,22,23,24,25,26,27,28,29,30,31,32,33,34,36,37,38,39,40,41,44,46,47,48,49,50,51,52,53,54,58,59,60,62,63,64,65,66,67,68,69,70,71,72,73,77,78,79,80,82,83,84,89,90,91,92]. Abbreviations: NEC: necrotizing enterocolitis; AKI: acute kidney injury; GFD: graft failure disease; ACLF: acute-on-chronic liver failure; APAP-ALF: acetaminophen-induced acute liver failure; CHC: chronic hepatitis C; and ACS: acute coronary syndrome.

Studies on FABP as a disease biomarker that have been discussed indicate its broad potential, as summarized in Figure 2. Some studies have conducted comprehensive testing using adequate testing methods and data analysis. They have been able to provide important suggestions in understanding the relationship between FABP and pathological diseases, as well as other related factors based on the test results. In conducting these tests, the use of anti-FABP antibodies as a measurement method is currently the most appropriate effort to optimize the specificity of FABPs in detecting diseases. However, further development is still needed regarding detection methods to ensure that the specificity of FABPs can match or even exceed established biomarkers. Nevertheless, the sample size in some studies was deemed insufficient to strengthen the reported findings, as was the case in studies on the potential of L-FABP as a biomarker for NEC [5], hepatocyte injury in patients after liver resection [29,30], and liver injury in patients with CHC [32]. Some of the studies discussed are still conducted on subjects with an inadequate number and cannot yet represent the clinical population. In addition, some studies still focus on measuring the expression levels of FABPs but have not been able to explain the valid correlation related to the tested FABP’s contribution to the discussed pathological diseases, as was the case in studies on the potential of T-FABP as a biomarker for prostate cancer [92] and I-FABP as a biomarker for acute ischemic stroke [40]. Although the test results show their potential as significant biomarkers, they have not been able to identify FABP’s participation in the development of these diseases. Therefore, it is important to conduct research focusing on the analysis of FABP mechanisms and signaling in these diseases.

The development of FABPs as a disease biomarker still faces various challenges and limitations that are important to consider for the progress of future research. As previously mentioned, the contribution and signaling pathway of some FABP isoforms as biomarkers for certain diseases is still not clearly and comprehensively known. Although some studies have reported the association between FABP expression levels and the severity or prognosis of a disease, and the discovery of high FABP expression is often associated with its potential in detecting a disease; this cannot be the sole theoretical basis for its contribution to the occurrence of a disease. For example, in a study on the potential of E-FABP as a biomarker for cervical cancer, the relationship between E-FABP expression level and cervical cancer has not been clearly explained through which pathway, and the significance of E-FABP expression to the severity and prognosis of cervical cancer has also not been explained in detail [84]. Although the role of E-FABP in cervical cancer may be similar to its contribution to prostate and breast cancer, a clear pathway is still needed to be further understood. The same goes for studies on the potential of A-FABP and Il-FABP as biomarkers for colorectal cancer, where it is mentioned that the pathways of both FABPs to colorectal cancer are still not clearly known [60]. Therefore, some hypotheses about the potential of FABPs as disease biomarkers that have been discussed need further comprehensive research to strengthen the foundation for the functional and clinical use of these biomarkers in the future. Further research on this matter is not only able to strengthen the hypothesis of FABPs as disease biomarkers but also allows the opening of the potential of FABPs as a therapeutic target for diseases.

The ability of FABPs to compete with other biomarkers to become a disease biomarker is also another challenge that needs to be considered. Based on the findings that have been discussed, some FABPs have shown good significance and are able to match the capabilities of existing biomarkers, such as L-FABP as a biomarker to distinguish patients with progressive AKI and cirrhosis, which has comparable abilities to NGAL, IL-18, and albumin as other candidate biomarkers [26]. In Pyati et al.’s study (2015), H-FABP even had the best detection ability compared to CK-MB and myoglobin [49]. However, it is important for us to consider the contributions of FABPs in the development of a disease as discussed earlier. Disease biomarkers that have been approved and used medically to diagnose diseases have a significant role in the development of diseases associated with them. For example, in myocardial injury, the detected biomarker for diagnosis is generally cTn (cardiac troponin). Since 2000, cTn has been recognized as the best biomarker for myocardial injury by the American College of Cardiology and The European Society of Cardiology in terms of sensitivity and specificity [93,94,95]. The presence of these biomarkers in a disease has been proven to provide significant contributions and can be a strong basis for observing the development and severity of the disease. Some studies also reported that the sensitivity of FABPs tends to be higher, but their specificity tends to be lower than existing biomarkers. For example, I-FABP in Shi et al.’s study (2015) showed higher sensitivity values compared to D-lactate as a biomarker for intestinal ischemia, although its specificity still needs to be optimized [38]; a similar case would be H-FABP as an ACS biomarker compared to hs-TropT [46]. Therefore, some FABPs cannot yet be used as a single biomarker, but need to be combined with other biomarkers that have better specificity and require an optimization of the diagnostic sensitivity.

In its future clinical use, the development of FABPs as a disease biomarker also faces challenges related to methods that can detect with optimal specificity and accuracy. FABPs, as a protein family with sequences and structures that are quite homologous and conserved between one isoform and another, as well as their widespread expression, pose a challenge in developing testing methods that can select and detect each FABP with a high level of confidence and fast testing time. Based on the findings that have been discussed, the testing methods used in research mostly are ELISA and immuno-turbidimetric assay methods. Currently, ELISA kits and immunoturbidimetric assay kits are available and include anti-FABP antibodies as FABP binding agents through the interaction of formed antigen–antibodies that are used to facilitate testing and measurement. The development of specific antibodies against each FABP can help increase the specificity of diagnosis using FABPs as biomarkers. The use of antibodies in the development of disease diagnostic methods has been widely used, such as COVID-19 [96,97], tuberculosis [98,99], and others. However, the development of antibodies against metabolic diseases may be much more difficult and challenging. Although the accuracy of the test results is good enough and can provide the necessary data as diagnostic support, the effectiveness and practicality of the test are still not optimal when applied in the future. Some of the studies discussed have used the principle of measurement and testing using anti-FABP antibodies. For example, Naruse et al. used a latex-enhanced immunoturbidimetric assay with mouse anti-human L-FABP monoclonal antibody-coated latex based on the antigen–antibody interaction that occurs through testing to measure L-FABP in urine and blood samples for AKI detection [24]. Another study by Teunissen et al. (2011) used their own developed polyclonal antibody anti-human B-FABP [91]. Further research and the assessment of the development of specific antibodies against an FABP isoform, whether in the form of polyclonal, monoclonal, or antibody fragments, could be a consideration for future researchers.

**Figure 3:**
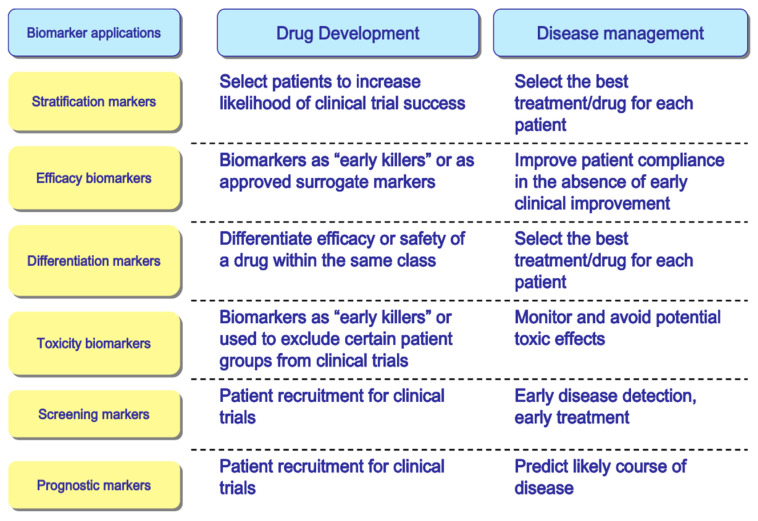
Classification of biomarker types based on their application in drug development and disease management. Source: OECD Report on “Policy Issues for the Development and Use of Biomarkers in Health” (2011) [101].

**Figure 4:**
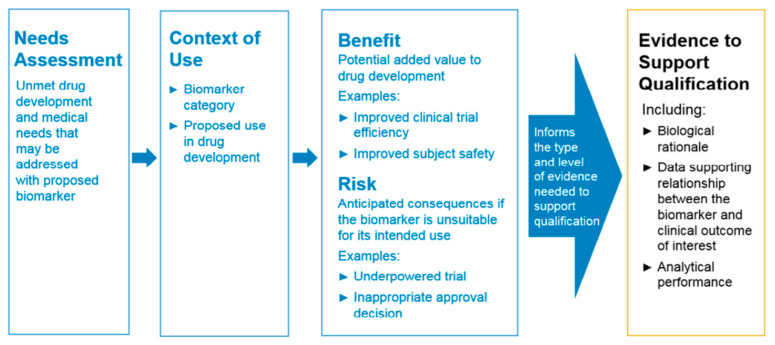
Evidentiary framework for biomarker qualification [100].

‘Biomarker’ itself is a broad and ambiguous term. According to the FDA, a biomarker is an objective measure that can be used to indicate a disease, health, or response to exposure or intervention. It is a variable that can be characterized and measured accurately. Biomarkers can be identified for their application in drug development, as well as disease management (Figure 3). The studies discussed so far still focus on the potential of FABPs as prognostic and screening biomarkers in disease management, where the reported findings refer to the ability of FABP to predict the progression of a patient’s disease and detect the occurrence of a disease in patients with specific symptoms. In its development for use as a clinical disease biomarker, there are qualifications that must be met. These qualifications have been summarized in Figure 4 based on the evidentiary framework guidance for biomarker qualification by the Food and Drug Administration (FDA). The fulfillment of these qualifications determines the suitability and validity of a biomarker to be used clinically in a legal and safe manner. In general, there are four stages in the qualification of a biomarker candidate: (1) identifying the urgency of using the biomarker for the disease in question, (2) describing the context of use in the form of the biomarker category and the proposed biomarker’s objectives, (3) defining the potential benefits that will be achieved if the proposed biomarker is qualified for use, and (4) defining the potential risks associated with the clinical use of the biomarker. The qualification of candidate biomarkers in their clinical use must be supported by supporting evidence, including biological rationale and data supporting the clinical correlation between the biomarker and the disease, as well as analytical performance that meets the standards [100]. Therefore, the studies discussed can refer to these qualifications as considerations for the validity of the reported hypotheses and serve as a basis for further research.

In conclusion, the findings related to the potential of FABPs as biomarkers for disease diagnosis and prognosis have been discussed. The conducted studies have proposed testing FABPs as disease biomarkers both in vitro and in vivo. Although most of the data indicate good significance, the role and contribution of some FABPs to the pathological diseases associated with their detection potential have not been extensively studied and understood. Therefore, further research is still needed to confirm and strengthen the reported findings. Exploring this area will not only reinforce existing hypotheses but may also generate new hypotheses regarding the potential of FABPs as therapeutic targets. The use of FABPs as disease biomarkers is often hindered by the challenges of specificity, which have not yet matched the existing biomarkers, despite their higher sensitivity, as reported in most literature. The development of rapid, accurate, and specific FABP analysis methods is recommended for future researchers to consider the clinical use of FABPs as disease biomarkers.

## Abbreviations

**Abbreviations**	**Meaning**
FABP	Fatty-acid-binding protein
FA	Fatty acid
MUFA	Monounsaturated fatty acid
PUFA	Polyunsaturated fatty acid
ELISA	Enzyme-linked immunoabsorbent assay
NEC	Necrotizing enterocolitis
AKI	Acute kidney injury
ACLF	Acute-on-chronic liver failure
APAP-ALF	Acetaminophen-induced acute liver failure
CHC	Chronic hepatitis C
AIS	Acute ischemic stroke
MI	Myocardial injury
AMI	Acute myocardial injury
ACS	Acute coronary syndrome
MACE	Major adverse cardiovascular events
PAD	Peripheral arterial disease
CAD	Coronary arterial disease
AD	Alzheimer’s disease
CSF	Cerebrospinal fluid
EC	Endothelial cell
HCC	Hepatocellular carcinoma
IBABP	Ileal-bile-acid-binding protein
